# Histochemical Characterisation and Gene Expression Analysis of Skeletal Muscles from Maremmana and Aubrac Steers Reared on Grazing and Feedlot Systems

**DOI:** 10.3390/ani11030656

**Published:** 2021-03-02

**Authors:** Giulia Foggi, Francesca Ciucci, Maria Conte, Laura Casarosa, Andrea Serra, Elisabetta Giannessi, Carla Lenzi, Stefano Salvioli, Giuseppe Conte, Marcello Mele

**Affiliations:** 1 Dipartimento di Scienze Agrarie, Alimentari e Agro-ambientali, University of Pisa, 56124 Pisa, Italy; francesca.ciucci88@gmail.com (F.C.); laura.casarosa@unipi.it (L.C.); andrea.serra@unipi.it (A.S.); giuseppe.conte@unipi.it (G.C.); marcello.mele@unipi.it (M.M.); 2 Dipartimento di Medicina Specialistica, Diagnostica e Sperimentale, University of Bologna, 40126 Bologna, Italy; m.conte@unibo.it (M.C.); stefano.salvioli@unibo.it (S.S.); 3 Centro di Ricerche Agro-Ambientali “Enrico Avanzi”, University of Pisa, 56122 Pisa, Italy; 4 Dipartimento di Scienze Veterinarie, University of Pisa, 56124 Pisa, Italy; elisabetta.giannessi@unipi.it (E.G.); carla.lenzi@unipi.it (C.L.)

**Keywords:** rustic cattle breed, grazing activity, atp1a1, mt-atp6, capn1 genes, skeletal fibres frequency, myosin ATPase

## Abstract

**Simple Summary:**

Muscle fibre types and sizes are important factors affecting muscle growth potential and meat quality. Their variability depends on some factors like muscle type, animal breed, physical activity, and they could be going through morphological or metabolic modifications, throughout animal life. Two muscles from Maremmana, an autochthonous breed from Tuscany (Italy), was compared to those from Aubrac, a breed from the Massif Central (France), under histochemical and gene expression points of view. Both these breeds were poorly studied, and the results identified Maremmana muscles were more oxidative in comparison to Aubrac. Moreover, steers of each breed were proportionally divided and reared on grazing or feedlot systems. Conversely to what was expected, the voluntary physical activity on pasture, another aspect poorly studied, influenced neither histochemical characteristics nor the gene expression.

**Abstract:**

This study aimed to characterise the fibre composition of *Triceps brachii* (TB) and *Semimembranosus* (SM) muscles from 20 Maremmana (MA) and 20 Aubrac (AU) steers, and the effect of grazing activity in comparison with feedlot system. The histochemical method was performed with the m-ATPase method with an acid pre-incubation, thus allowing to distinguish type I, IIA, and IIB fibres. Additionally, on total RNA extracted from SM muscle, the expressions of atp1a1, mt-atp6, and capn1 genes were evaluated, in order to find potential associations with muscle fibre histochemical characteristics. In SM muscle, the MA steers had the greater frequency of oxidative fibres (type I and IIA) and the higher atp1a1 expression, in comparison to AU steers. Conversely, AU steers had a greater frequency of type IIB fibres, and the higher capn1 expression. A similar histochemical pattern was observed in TB muscle. The grazing activity was probably insufficient to determine differences both for fibre proportion and size, and gene expressions, except for mt-atp6 expression that was surprisingly highest in feedlot MA in comparison to other steers. These findings further the knowledge of muscle properties belonging to these breeds, and the effect of voluntary physical activity since few studies were available in this regard.

## 1. Introduction

It is well established that skeletal muscle tissue is composed of at least three types of muscle fibres. The fibre type diversities are based on differences in contractile, metabolic, morphological, and physiological features, as mainly summarized in Table 1. Based on these features, muscle fibres are classified accordingly to different systems. The classification based on histochemical analyses, as defined by Brooke and Kaiser [1], identified type I, IIA, and IIB fibre, while that based on enzyme activity classified slow-twitch oxidative (SO), fast-twitch oxidative glycolytic (FOG), fast-twitch glycolytic fibres (FG). The skeletal muscle fibre characteristics and classifications were largely reviewed in humans [2,3] and small and large animals [4,5,6,7].

Throughout the animal life, exercise, diet, temperature, weight, age, and growth-promoting agents affect the physiological balance of muscle tissue and then fibre characteristics [8]. All these factors are responsible for the quantitative mechanism of regulation that causes changes in fibre size and muscle mass. Moreover, a qualitative mechanism is responsible for fibre type changing by following a specific pathway I ⇆ IIA ⇆ IIB and mainly due to MyHC polymorphism [2]. Within a species, breed and genotype are the main factors affecting fibre type composition of a given muscle [6].

During the last decades, the genetic improvement of cattle breeds has been mainly driven by the meat industry’s interest toward the increase of average daily gain, feed conversion rate, slaughtering performance, and the improvement of meat quality. The meat industry is thereby pushing to select genetic types with larger fibres (type II) and muscle with higher growth potential [5,9,10]. Thus, some rustic bovine breeds with lower productivity, but greater adaptability to specific environmental conditions, are less farmed, while they might own interesting features to sustain production and to cope with climate change [11]. The associations between muscle fibre characteristics and meat quality aspects have been previously reported, concerning the meat colour [12], the content of intramuscular fat [13], the tenderness [7], or the water holding capacity [14]. Therefore, detailed characterisation of muscle fibre is aimed to explore the potential of rustic breeds in meat production.

In humans [15,16], rats [17], and more rarely in cattle [18], the expression of genes encoding for some ATPase were related to the energy metabolism of the cells, and studied in associations with muscle properties. In this study, atp1a1 and mt-atp6 encoding genes were considered as representative for two ATPase otherwise located. The former encoded for the subunit α1 of Na^+^-K^+^-ATPase enzyme located in membranes, where it exerted the catalytic and basal pump activity functions of the enzyme [19]. The second is a mitochondrial gene encoding for the subunit 6 of the ATPase involved in the final step of oxidative phosphorylation pathway [18]. Moreover, the capn1 gene that encoded for the large subunit of µ-calpain protease was also considered as an important proteomic biomarker for the tenderization process [20].

This study aimed to characterise the fibre types of *Triceps brachii* (TB) and *Semimembranosus* (SM) muscles from two rustic bovine breeds (Maremmana and Aubrac), within two rearing systems (grazing and feedlot). Additionally, the possible connections between the expression of the three genes and the histochemical characteristics were explored by considering the SM muscle.

## 2. Materials and Methods

### 2.1. Animals and Experimental Design

A total of 40 steers, 20 Maremmana (MA) and 20 Aubrac (AU) of 4.5-months-old and with an initial average body weight of 250 ± 51 kg, were included in the study. Animals of each genetic type were randomly allotted in two experimental groups (10 per group) in function of the rearing system: pasture or feedlot. Every ten animals’ group was randomly distributed into two subgroups of three steers and one subgroup of four animals, in order to obtain three replicates for each rearing system and each breed, with a total of twelve subgroups. Every feedlot area was 2500 m^2^, whereas every pasture area was approximately 10 ha. The feedlot and the pasture areas, all located in southern Tuscany (Italy), were equally subdivided into three lots in order to obtain three replicates of every ten animals’ group. MA is an autochthonous breed of this area, and grazing is the typical production system for this breed. In the same area, MA is reared in the feedlot system too. AU breed is not typically reared in this area, however, being AU cattle, a rustic breed from Massif Central (France), it is suitable to be reared on pasture.

Concerning the diets given to the steers, both feedlot and pasture groups received grass hay ad libitum. In addition, the feedlot groups received 1 kg of concentrate mixture every 100 kg of live weight per head per day and the grazing groups received grass hay available on pasture and the concentrate mixture only when the grass availability was insufficient. The amount of concentrate administered to grazing steers was monthly decided to obtain similar individual average daily gain. The individual average daily gain recorded (0.78 kg/d expressed as a mean over the four groups throughout the experimental period) was not significantly different. In any case, the maximum amount of concentrate daily administered was not greater than 1 kg every 100 kg of live weight per head. The concentrate was administered twice a day. All the animals had free access to shade and water.

The steers were slaughtered between 20–22 months of age when they reached a weight of about 600 kg, which is a typical slaughter weight in the southern Tuscany area.

### 2.2. Muscle Sampling

At slaughter, within 20 min after the killing procedure, whole TB and SM muscles from the right side of the carcass were collected. In the meanwhile, temperature and pH of muscles were measured.

For histochemical analysis, cubic samples (0.5 cm^3^) were dissected from each muscle and immediately frozen in liquid nitrogen for 30 s, then stocked at −80 °C.

In order to prevent cells from cold damages, each sample was soaked in a histology gel (Tissue-Tek^®^ O.C.T., Sakura Finetek Europe, Alphen aan den Rijn, The Netherlands) in a small open container.

### 2.3. Histochemical Analysis

**Figure 1:**
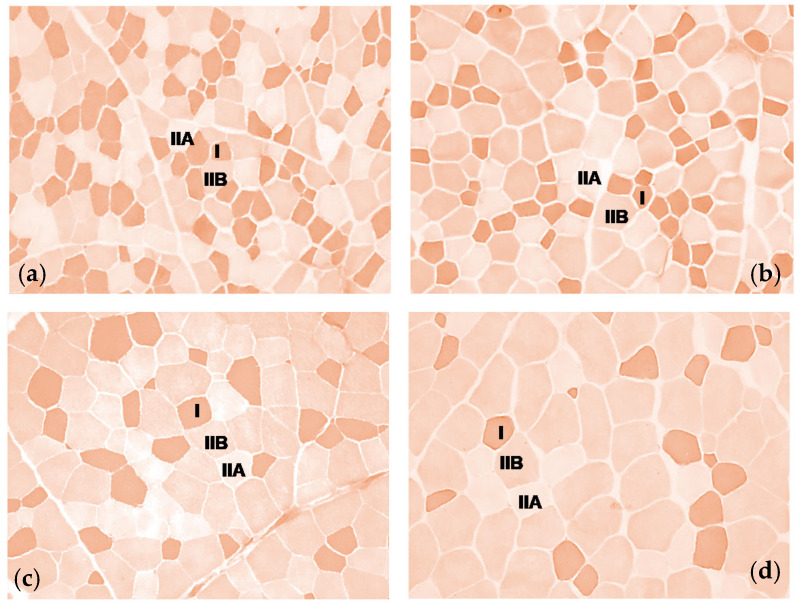
Histochemical m-ATPase staining after acid pre-incubation at pH 4.45–4.47 of: (**a**) *Semimembranosus* (SM) of Maremmana (MA); (**b**) *Triceps brachii* (TB) of MA; (**c**) SM of Aubrac (AU); (**d**) TB of AU, using a 10× objective.

The frozen muscle tissue transverse sections of 10 μm thickness were obtained with a cryostat (Leitz 1720, Leitz, Wetzlar, Germany) at −25 °C. Throughout storage and during the cutting of histological sections, the temperature was kept under −25 °C in order to prevent protein denaturation and activity loss of myofibrillar adenosine triphosphatase (m-ATPase). The histochemical method was performed in accordance with that reported by Brooke and Kaiser [1,21] with pre-incubation in sodium acetate buffer at pH values adapted to bovine tissue (pH = 4.45–4.47). Type I fibres were the darkest, type IIB were intermediate, and type IIA were the lightest, as shown in Figure 1. Each solution was freshly prepared, and each step was done at room temperature.

### 2.4. Image Analysis

Microscope field images were obtained through an optic microscope (Nikon^®^ Eclipse, Nikon Instruments, Calenzano, Italy) equipped with a digital images acquisition and elaboration system (Nikon^®^ NIS elements BR Microscope Imaging Software, Nikon Instruments, Calenzano, Italy), using a 10× objective. Four images were randomly collected from two slides of each muscle, for a total of eight images acquired per animal. The outlines of each muscle fibre were marked out manually and the fibre type was distinguished according to the colour, in order to obtain the cross-sectional area (CSA) of each muscle fibre, and expressed as µm^2^ (Figure 1). The mean fibre area per sample and the percentage area (% CSA) for each fibre type per sample were calculated. At least 300 fibres per sample unit were analysed.

### 2.5. Gene Expression Analyses

Total RNA from SM muscle was extracted using mirVANA kit (Ambion, Thermo Fisher Scientific, Waltham, MA, USA), according to the manufactures’ instructions. After DNase treatment with TURBO DNA-free kit (Ambion, Thermo Fisher Scientific, Waltham, MA, USA), RNA was quantified with NanoDrop (Thermo Fisher Scientific, Waltham, MA, USA). cDNA was synthesized using iScript™ cDNA Synthesis Kit (Bio-Rad Laboratories, Inc., Hercules, CA, USA) according to the manufacturer’s instructions. Relative quantification was performed by real-time RT–PCR by using iTaq™ Universal SYBR Green Supermix (Bio-Rad Laboratories, Inc., Hercules, CA, USA) and Rotor Gene Q 6000 system (QIAGEN GmbH, Hilden, Germany). All data were normalized to GAPDH expression. All oligonucleotide pre-designed primers were from Bio-Rad. All information on these primers is available at website www.bio-rad.com/PrimePCR (accessed on 19 February 2019). Real time RT-PCR reactions were performed in duplicate in the same run and each run was repeated twice for all measurements. The mean of experiments was considered for the analysis. All reactions consisted of an initial denaturation step at 95 °C for 2 min, followed by 40 cycles of 95 °C for 5 s and 60 °C for 30 s. The referent sample, used as an internal calibrator in each run, resulted from a pool of all feedlot samples. The ΔCt value was calculated by subtracting the Ct value for housekeeping gene from the Ct value for the target gene of the same sample. The ΔΔCt was then calculated by subtracting the ΔCt value of the referent sample (pooled samples) from the ΔCt value of the subject. Relative expression level was then determined by calculating 2-ΔΔCt.

### 2.6. Statistical Analysis

Data were analysed by the following linear model (SAS Institute Inc., Cary, NC, USA, 2010):

Yijz = μ + Bi + Rj + Bi x Rj + Ak + εijz(1)

where Yijz = variables (frequency, CSA, % CSA); μ = overall media, Bi = fixed effect of the i^th^ breed (AU and MA); Rj = fixed effect of the j^th^ rearing system (feedlot and pasture); Bi × Rj = fixed effect of interaction between breed and rearing system; Ak = random effect of the k^th^ animal; εijz = random error.

Data were independently analysed for each muscle considered: SM and TB muscle. Differences were declared significantly different at *p*-value < 0.05. When interaction effect was significant, a post hoc Tuckey’s analysis was done.

## 3. Results and Discussion

### 3.1. Type I, IIA, IIB Fibre Frequencies

In TB muscle, type IIA and IIB frequencies were significantly different between AU and MA steers (*p* < 0.01), as reported in Table 2. Type IIA frequency was higher in MA muscles, and type IIB frequency was higher in AU muscle composition. A similar pattern was observed for SM muscle, where type IIA was different only in tendency (*p* = 0.06). the type I frequency tended to be higher in both MA muscles if compared with AU (*p* = 0.06).

To the best of our knowledge, the MA histochemical characteristics were not previously analysed with myosin histochemical ATPase method. However, muscles from other rustic bovine breeds such as *Longissimus dorsii* (LD) from Alentejana had a similar fibre composition (mean frequencies between discontinuous and continuous growth system: 31.3% I, 29.7% IIA, 38.9% IIX [22]) to what was observed in SM muscles from MA steers (30% I, 29% IIA, 41% IIB).

Conversely, muscle fibre composition of AU cattle was previously studied by Jurie et al. [23] and [24]. In these studies, Aubrac bulls from 15 to 24 months and cull cows from 4 to 9 year were considered, respectively. In both studies, the authors classified fibres as fast oxidative (FO), fast oxidative glycolytic (FOG), and fast glycolytic (FG). Although the fibres were classified according to a different system, the results of type IIA and IIB fibre frequencies obtained in the present study (22.5% IIA; 56.8% IIB, averages over the two muscles and rearing systems) were consistent with FOG and FG fibre frequencies obtained in the study from Jurie et al. [23] (20.9% FOG; 53.0% FG, averages of three muscles of AU bulls). With regard to the type I fibre, the comparison with the same study (20.7% type I vs. 26.2% SO [23]) showed a slight difference. The above-mentioned difference might be related to a different rearing system, different fibre classification systems adopted or, most likely, because of the different muscles considered in the average calculation. However, a partial incompatibility of muscle fibre classifications is also well documented [2,3,25], and the histochemical m-ATPase technique cannot distinguish hybrid fibres, since they have intermediate pH stability if compared with the pure fibres [6]. In the study of Picard et al. [25], they compared the m-ATPase histochemical technique with monoclonal specific antibody staining in bovine muscles. Type I and IIB fibres were classified identically, while type IIAB that was about 10% of the total were identified as type IIA fibres, causing an overvaluation of the latter. For our purpose, the identification of type IIAB as type IIA fibres does not give more insights into the metabolic features, having both these fibres oxidative and glycolytic capacity, as reported in Table 1.

Concerning muscle fibre composition in comparison to other cattle breeds and according to literature, muscle fibre composition of AU steers was similar to that of cosmopolite beef breeds. For example, Jurie et al. [23] reported no significant difference in muscle fibre composition between Aubrac (26.2% SO, 20.9% FOG, 53.0% FG), Limousin (25.6% SO, 19.0% FOG, 55.5% FG), and Charolais (27.8% SO, 19.3% FOG, 52.9% FG) bulls. The SM composition of the AU steers from the present study (20.1% I, 23.3% IIA, 56.6% IIB) was very close to that reported by McGilchrist et al. [26] for low-muscling Angus steers (21.1% I, 25.7% IIA, 51.9% IIB).

MA steers had a unique fibre composition (28.8% I, 29.2% IIA, 41.9% IIB, averages over the muscles and rearing system) in comparison with the other breed reported above. Particularly, MA muscles had less glycolytic fibres if compared with AU and other cosmopolite beef breeds, as reported above. According to Hwang et al. [27], type I and type IIB fibres are inversely correlated to beef quality: beef with higher type I frequency and lower type IIB frequency had higher tenderness and marbling. On the contrary, Gagaoua et al. [20] reported that tenderness was positively associated with the myosin heavy chain (MyHC-IIX), mainly contained in type IIB fibres. Many reviews reported these controversial results, by suggesting that other factors, such as connective tissue and intramuscular fat content, affect meat tenderness [5,6,7]. Further researches are needed to assess any type of correlation between meat quality and histochemical features in AU or MA beef.

The life-long voluntary activity normally induces a transition (fibre plasticity) from IIB (glycolytic) → IIA → I (oxidative) fibres, depending on type and duration of physical activity and the involvement level of muscles [8,28]. Despite a lower frequency of type IIB, fibres in muscles from grazing were expected. Our findings showed no significant effect of grazing activity on fibre frequencies both in TB and SM from AU and MA (Table 2). Only greater type IIA frequency, with no significative differences (*p* = 0.09), were scored in TB muscles of grazing steers. According to Gangnat et al. [29], a higher significative frequency of type IIA fibre was scored in muscles (*Longissimus thoracis*, *Biceps femoris*) of suckling calves after a period of 11 weeks on flat pasture in comparison with steep pasture, while the opposite was observed for type IIB fibres. Vestergaard et al. [30] found a lower frequency of type IIB fibres in muscles (*Semitendinosus*, *Longissimus dorsi*, *and Supraspinatus*) from Friesian young bulls reared on an extensive system in comparison with the intensive system. Even though a few other studies revealed differences induced by physical activity, in the present study, the 15 months-minimum period of steers on pasture or the intensity of physical activity were probably insufficient to determine significative differences in histochemical muscle features between pasture and feedlot steers. Several authors have generally focused the study of histological fibre features in bovine species on feeding regimen [31,32,33], slaughter weight [34], growth, and breed [23,35] changes. To the best of our knowledge, in cattle, the effect of physical activity on muscle fibres is less documented, while for other species, mostly human and rat, more data are available.

Further studies on MA and AU are needed in order to assess other effects related to muscle plasticity, such as the studies of the activities of oxidative or glycolytic enzymes. On the other hand, an enhanced effect of oxidative enzyme activity level in TB muscle was already found in bulls kept under a dietary restricted regimen, with unaltered muscle fibre proportion [31].

### 3.2. Cross-Sectional Area (CSA) of Type I, IIA, IIB Fibre

AU and MA steers had similar muscle fibre size, independent of the muscle considered and fibre type (Figure 1). To the best of our knowledge, the CSA of MA bovines were measured only once in the study of Serra et al. [10]. In their study, they considered fibres from TB and SM muscles of grazing female MA slaughtered at 19 months. Contrary to what expected, they recorded a higher mean of fibres CSA values (5472 µm^2^, average over TB and SM muscles), in comparison to those measured in the present study (3234 µm^2^, average over TB and SM muscles), irrespective of the muscle considered. Schreurs et al. [36] reported in a meta-analysis the relationship of CSA with the degree of maturity in heifers, bulls, and steers: CSA increased quadratically and linearly in bulls and cows, respectively (effect of sex), and bulls had a greater linear coefficient rather than steers. The castration effect negatively affected hypertrophy during growth, probably because of the different testosterone production [31,36].

In the study of Jurie et al. [23], fibre CSAs of AU bulls were compared to other breeds, such as Limousin, Charolais, and Salers bulls, with the result that breed significantly affected CSA. Unfortunately, the muscle fibre CSA of AU from that study (3187 µm^2^ SO, 3870 µm^2^ FOG, 5467 µm^2^ FG [23]) was difficult to compare to ours, probably mainly because they reported the CSAs as average over ages (from 15 to 24 months) and over three different muscles (*Longissimus thoracis*, *Semitendinosus*, and TB). Equally, Jurie et al. [24] measured CSA from the same muscles but collected them from cull cows slaughtered from 4 to 9 years of age. The CSA values of AU cull cows (2428 µm^2^ SO, 2993 µm^2^ FOG, 4227 µm^2^ FG) were, in general, similar to ours (2732 µm^2^ I, 2713 µm^2^ IIA, 4337 µm^2^ IIB, averages over muscles and rearing systems).

McGilchrist et al. [26] measured CSAs of SM muscles collected from steers slaughtered around two years of age, as we did. Consequently, similar CSA values were expected, although they considered Angus steers. Their results for type IIA and IIB CSA values of high muscling steers (2674 µm^2^ IIA, 4518 µm^2^ IIB) were similar to those we scored for AU and MA SM muscle (2978 µm^2^ IIA, 4637 µm^2^ IIB, and 3277 µm^2^ IIA, 4693 µm^2^ IIB, respectively), independently to the rearing system considered. With regard to type I CSA, those from high-muscling Angus were much larger (3224 µm^2^) in comparison to AU (2254 µm^2^) or MA fibres (2239 µm^2^), by suggesting an enhanced oxidative potential in high muscling Angus steers, which was already found by comparing high vs. low muscling steers [26].

All muscle fibre types from MA and AU steers reared on pasture scored higher CSA values in comparison with feedlot steers, but without significant difference. In SM muscle, rearing system affected only in tendency type IIB CSA (*p* = 0.08). In accordance with our results, Vestergaard et al. [30] by comparing Friesian bulls in extensive vs. intensive systems did not find any significative difference among CSA values of *Longissimus dorsii* muscle. On the contrary in the same study, *Semitendinosus* and *Supraspinatus* muscle from bulls on the extensive system had larger CSA values for type IIA and IIB fibres, suggesting in these muscles a higher proportion of variation of CSA related to physical activity. In a more recent study by Gangnat et al. [29] and accordingly to our results, CSAs from suckling calves did not change after a period of 11 weeks on steep pasture in comparison to flat pasture, neither in *Longissimus thoracis* nor in *Biceps femoris* muscle fibres. As previously noticed by Gangnat et al. [29], still a few studies about the effect of physical activity on cattle muscle exist, and further research is needed to understand the adaptations of the various types of bovine muscle varying the rearing system.

### 3.3. Percentage of Cross-Sectional Areas

Muscle characteristics varying depending upon muscle type and sexes [36], and fibre characteristics of a given muscle is mainly influenced by age [35], genotype [32], selection [26,37], physical activity (as reviewed in human by Fry AC [28]), and to a lesser extent by feeding regimen [32,33]. Since the number of muscle fibres of a selected muscle is almost set prenatally, the hypertrophy of fibres is inversely correlated with the number of muscle fibres [9]. Thus, fibre plasticity and hypertrophy influence life-long fibre frequencies and fibre CSA modifications. CSA percentage allowed to simultaneously assess the effects of both frequency and CSA of each fibre type of a given muscle. Being the CSA of each fibre type both in TB and SM not significantly modified by the grazing activity or breed, the values of % CSA followed the results obtained for fibre frequencies. In both muscles, % CSA of type IIA and IIB fibres were higher in MA and AU, respectively (*p* ≤ 0.001 TB; *p* < 0.05 SM, Table 2). Moreover, type I % CSA tended to be higher in MA muscles in comparison to AU (*p* < 0.10).

To the best of our knowledge, any previous study reported % CSA values for MA breed. Our results suggested that MA muscles might have higher oxidative potential or lower glycolytic potential, in comparison to AU or other cattle breeds. In the present study, MA had less than 55% CSA of glycolytic fibres and the remaining 45% CSA of oxidative fibres (average calculated over TB and SM muscles for IIB fibres), while AU had almost the 70% IIB CSA. Similarly to AU, in other breeds, it was reported a value of % CSA of glycolytic fibres mainly higher than 60%, such as in Charolais, Limousin, and Salers bulls (averages over three muscles [23]); Angus steers (*Semimembranosus* muscle [26]); Montbéliard steers (*Longissimus dorsii* muscle [31]). Conversely, Alentejana bulls, a non-specialised beef breed from Portugal, had even less than 50% CSA IIB in *Longissimus dorsii* muscle, suggesting a higher oxidative potential contrary to the breeds above-mentioned [22]. Further studies conducted on rustic bovine genotypes, such as Ma or Alentejana, could reveal a relationship between the lower % CSA of type IIB fibre and oxidative/glycolytic potential of the muscle and, consequently, some related meat-quality aspects.

With regard to rearing system, it did not affect the % CSA of any fibre types, neither in TB nor in SM muscle.

### 3.4. Gene Expression in SM Muscle

The expressions of two genes representative for two ATPase otherwise located (atp1a1, mt-atp6) and a gene representative for the calpain system (capn1) were evaluated in SM muscle (Table 2), in order to find potential associations with muscle fibre histochemical characterisation of the two bovine breeds considered in the present study.

Atp1a1 gene encoded for subunit α1 of Na^+^-K^+^-ATPase enzyme. To the best of our knowledge, this is the first study reporting the atp1a1 expression of bovine skeletal muscle tissue. Previously, the atp1a1 expression was investigated in cattle in blood samples, and in order to correlate the Na^+^-K^+^-ATPase activity with the animal response on heat stress [19,38]. The MA steers had a higher expression of atp1a1 gene in comparison to AU, suggesting a potential association with muscle fibre proportion that differed between MA (higher oxidative fibre frequency) and AU (higher glycolytic fibre frequency). According to our assumption, a previous study conducted on rat skeletal muscle found a positive correlation between oxidative muscle type and α-1 subunit expression [17]. With regard to physical activity, unexpectedly, the rearing system did not affect atp1a1 expression. In fact, a previous study conducted on humans reported that submaximal exercise increased α-1 subunit expression after 24 h, without affecting α-1 protein abundance [39]. As previously hypothesised for CSAs, voluntary physical activity on pasture was probably insufficient to induce modifications also on atp1a1 expression.

The mitochondrial gene mt-atp6 encoded for the subunit 6 of the ATPase, and is involved in the final step of oxidative phosphorylation pathway. Its expression was the highest in feedlot MA compared to the other three groups (*p* < 0.05). Histochemical characteristics would not completely justify the possibly enhanced oxidative metabolism in feedlot MA, particularly in comparison with pasture MA, that had similar muscle fibre proportion and fibre size. In a previous study conducted on Avileña-Negra Ibérica beef cattle breed, the mt-atp6 was up-regulated in the muscle (*Psoas major*, 29% I, 21% IIA, 35% IIB) containing the type IIB fibres rather than the *Flexor digitorum* muscle that did not contain the type IIB [18]. It is interesting to note that the two muscles differed for glycolytic (higher in *Psoas major* being type IIB fibres present) but not for oxidative activity. Their findings and our results suggested that the level of mt-atp6 expression could not be predicted either with oxidative activity level or with muscle fibre proportion.

Capn1 encoded for the large subunit of µ-calpain protease, which is involved in the tenderisation process. According to Mberema et al. [40], tenderness could be, more generally, modulated through the differential expression of calpain system gene expressions, mainly µ-calpain (CAPN1), calpain3 (CAPN3), and calpastatin (CAST).

In the present study, the SM muscle of AU had significantly higher Capn1 gene expression, suggesting that AU beef could be tender in comparison to MA muscle. However, it should also be considered that capn1 could only partially affect tenderness. AU muscle had a larger proportion of glycolytic fibre in comparison to MA (+16% IIB) that impacts on pH value from slaughtering and the tenderisation process, afterwards. Moreover, gene expression is not necessarily related to µ-calpain activity. In a previous study, the µ-calpain was compared at activity level among two cattle breeds, Angus and Simmental steers, and no difference was found [41].

To the best of our knowledge, any previous study reported the effect of rearing system on capn1 gene expression, while the influence of feeding strategies was reported only once by Coria et al. [42]. In their study, supplementing pasture-finished steers with corn silage determined a down-regulation of calpain 1 and 2. In another study, feeding restriction in female calves was associated with lower calpain activity and higher calpastatin expression [43]. In the present study, any effect was found by comparing the feedlot vs. pasture system.

## 4. Conclusions

Both SM and TM muscles from MA and AU steers were mainly distinguishable by fibre composition: MA had the larger proportion of oxidative fibres (type I, type IIA) and AU of glycolytic fibres (type IIB). The muscle fibre proportion of MA suggested being associated with the higher atp1a1 expression, while that of AU to the higher capn1 expression. However, the grazing activity did not significantly affect either the histochemical properties considered or the atp1n1 and capn1 gene expressions. Only Feedlot MA steers had the highest mt-Atp6 expression compared to other steers, but histochemical characteristics need to be integrated with other studies to understand this divergence. These results furthered the knowledge of these breeds less genetically improved and less studied, and it might be useful to broaden the genetic improvement strategies. Also, the effects of training on histochemical properties and gene expression in cattle breeds need to be furthered at transcriptomic and metabolic levels, since the grazing physical activity seem to be insufficient to induce modifications both on histochemical properties and gene expressions.

## Figures and Tables

**Table 1:** Main morphological, metabolic, and histochemical features of muscle fibre [1,4,6] ^1^.

Fibre Types	Type I/SO	Type II A/FOG	Type II B/FG
**Morphological Features**			
Cross-sectional area	small	intermediate	large
Mitochondrial number	high	high	low
Intramuscular fat	high	low	low
**Metabolic Features**			
Contraction speed	low	high	high
Fatigue resistance	high	intermediate	low
ATP production pathway	aerobic	aerobic/Anaerobic	anaerobic
Mitochondrial ATPase activity	high	low/high	low
Myoglobin content	high	intermediate	low
Glycogen	low	intermediate	high
**Contractile Features**			
Myosin ATPase activity	low	high	high
MyHC-II isoforms	MyHC-I	MyHC-IIA	MyHC-IIX
**Histochemical Analysis**			
m-ATPase, pH 9.4	white	dark	dark
m-ATPase, pH 4.5	red	white	medium dark

^1^ Acronyms used in the table: “SO” Slow oxidative; “FOG” Fast oxidative-glycolytic; “FG” Fast glycolytic.

**Table 2:** Effect of the breed (B), rearing system (R), and their interaction (B × R) on histochemical characteristics and gene expression in *Triceps brachii* (TB) and *Semimembranosus* (SM) muscles of AU and MA steers ^1^.

Item	MA		AU		SE	B	R	B × R
	Feedlot	Pasture	Feedlot	Pasture				
**TB**								
**% I**	25.56	29.94	22.97	19.55	3.36	0.06	0.89	0.26
**% IIA**	28.65	30.26	18.57	25.00	2.28	0.002	0.09	0.30
**% IIB**	45.76	39.79	58.51	55.52	3.90	<0.001	0.26	0.71
**CSA mean**	2827.34	2961.24	2844.89	3571.70	295.18	0.30	0.16	0.33
**CSA I**	1630.81	1817.82	1659.49	2072.68	186.51	0.45	0.12	0.55
**CSA IIA**	2621.28	2863.98	2229.42	2664.62	277.79	0.30	0.23	0.73
**CSA IIB**	3800.73	4045.38	3514.66	4556.24	427.06	0.79	0.14	0.36
**% CSA I**	14.89	17.89	13.69	11.22	2.04	0.07	0.90	0.19
**% CSA IIA**	26.31	28.74	14.49	19.84	2.91	0.001	0.19	0.62
**% CSA IIB**	58.81	53.39	71.84	69.50	3.63	<0.001	0.30	0.68
**SM**								
**% I**	27.24	32.82	19.83	20.39	5.01	0.06	0.55	0.62
**% IIA**	29.87	28.08	22.83	23.78	2.79	0.06	0.88	0.63
**% IIB**	42.91	39.10	57.37	55.82	5.86	0.01	0.65	0.85
**CSA Mean**	3207.09	3938.77	3704.33	3886.22	312.43	0.49	0.16	0.39
**CSA I**	1973.15	2504.12	2199.13	2310.86	268.23	0.95	0.25	0.44
**CSA IIA**	2999.54	3555.29	2928.75	3028.32	375.46	0.44	0.39	0.55
**CSA IIB**	4069.74	5316.97	4484.07	4791.39	416.30	0.90	0.08	0.27
**% CSA I**	18.00	21.91	11.68	13.34	4.21	0.09	0.52	0.79
**% CSA IIA**	28.28	25.28	19.01	19.07	3.49	0.04	0.68	0.67
**% CSA IIB**	53.72	52.79	69.33	67.58	6.80	0.04	0.85	0.95
**Gene Expression**								
**atp1a1**	3.75	3.14	0.60	1.21	0.43	<0.001	0.99	0.15
**mt-atp6**	3.60 ^A^	1.59 ^B^	0.68 ^B^	0.50 ^B^	0.31	<0.001	0.001	0.006
**capn1**	0.51	0.46	1.02	0.99	0.12	<0.001	0.74	0.89

^1^ Acronyms used in the table: “SE” Standard Error; “mean area” average of muscle fibre cross-sectional area, without considering the fibre types; “CSA” cross-sectional area; “% CSA” calculated percentage of cross-sectional area per fibre type; capitalized apexes “A”, “B” within a row mean significant differences with *p* < 0.01.

## Data Availability

The data presented in this study are available in the article.

## References

[1] Brooke M.H., Kaiser K.K. (1970). Muscle Fiber Types: How Many and What Kind?. Arch. Neurol..

[2] Pette D., Staron R.S. (1990). Cellular and Molecular Diversities of Mammalian Skeletal Muscle Fibers. Rev. Physiol. Biochem. Pharmacol..

[3] Bottinelli R., Reggiani C. (2000). Human Skeletal Muscle Fibres: Molecular and Functional Diversity. Prog. Biophys. Mol. Biol..

[4] Choi Y.M., Kim B.C. (2009). Muscle Fiber Characteristics, Myofibrillar Protein Isoforms, and Meat Quality. Livest. Sci..

[5] Lee S.H., Joo S.T., Ryu Y.C. (2010). Skeletal Muscle Fiber Type and Myofibrillar Proteins in Relation to Meat Quality. Meat Sci..

[6] Lefaucheur L. (2010). A Second Look into Fibre Typing—Relation to Meat Quality. Meat Sci..

[7] Picard B., Gagaoua M. (2020). Muscle Fiber Properties in Cattle and Their Relationships with Meat Qualities: An Overview. J. Agric. Food Chem..

[8] Lefaucheur L., Gerrard D. (2000). Muscle Fiber Plasticity in Farm Mammals. J. Anim. Sci..

[9] Rehfeldt C., Fiedler I., Dietl G., Ender K. (2000). Myogenesis and Postnatal Skeletal Muscle Cell Growth as Influenced by Selection. Livest. Prod. Sci..

[10] Serra A., Conte G., Giannessi E., Casarosa L., Lenzi C., Baglini A., Ciucci F., Cappucci A., Mele M. (2017). Histological Characteristics, Fatty Acid Composition of Lipid Fractions, and Cholesterol Content of Semimembranosus and *Triceps Brachii* Muscles in Maremmana and Limousine Bovine Breeds. Front. Vet. Sci..

[11] Rovelli G., Ceccobelli S., Perini F., Demir E., Mastrangelo S., Conte G., Abeni F., Marletta D., Ciampolini R., Cassandro M. (2020). The Genetics of Phenotypic Plasticity in Livestock in the Era of Climate Change: A Review. Ital. J. Anim. Sci..

[12] Klont R.E., Brocks L., Eikelenboom G. (1998). Muscle Fibre Type and Meat Quality. Meat Sci..

[13] Kirchofer K.S., Calkins C.R., Gwartney B.L. (2002). Fiber-Type Composition of Muscles of the Beef Chuck and Round. J. Anim. Sci..

[14] Joo S.T., Kim G.D., Hwang Y.H., Ryu Y.C. (2013). Control of Fresh Meat Quality through Manipulation of Muscle Fiber Characteristics. Meat Sci..

[15] Wyckelsma V.L., Perry B.D., Bangsbo J., McKenna M.J. (2019). Inactivity and Exercise Training Differentially Regulate Abundance of Na+-K+-ATPase in Human Skeletal Muscle. J. Appl. Physiol..

[16] Hundal H.S., Maxwell D.L., Ahmed A., Darakhshant F., Mitsumotoi Y., Klip A. (1994). Subcellular Distribution and Immunocytochemical Localization of Na,K-ATPase Subunit Isoforms in Human Skeletal Muscle. Mol. Membr. Biol..

[17] Thompson C.B., Dorup I., Ahn J., Leong P.K.K., Mcdonough A.A. (2001). Glucocorticoids Increase Sodium Pump A2- and Β1-Subunit Abundance and MRNA in Rat Skeletal Muscle. Am. J. Physiol..

[18] Moreno-Sánchez N., Rueda J., Carabaño M.J., Reverter A., McWilliam S., González C., Díaz C. (2010). Skeletal Muscle Specific Genes Networks in Cattle. Funct. Integr. Genomics.

[19] Liu Y., Li D., Li H., Zhou X., Wang G. (2011). A Novel SNP of the ATP1A1 Gene Is Associated with Heat Tolerance Traits in Dairy Cows. Mol. Biol. Rep..

[20] Gagaoua M., Terlouw C., Picard B. (2019). The Associations between Proteomic Biomarkers and Beef Tenderness Depend on the End-Point Cooking Temperature, the Country Origin of the Panelists and Breed. Meat Sci..

[21] Brooke M.H., Kaiser K.K. (1970). Three “Myosin Adenosine Triphosphatase” Systems: The Nature of Their PH Lability and Sulfhydryl Dependence. J. Histochem. Cytochem..

[22] Costa P., Simões J.A., Alves S.P., Lemos J.P.C., Alfaia C.M., Lopes P.A., Prates J.A.M., Hocquette J.F., Calkins C.R., Vleck V. (2017). Beef Palatability and Its Relationship with Protein Degradation and Muscle Fibre Type Profile in Longissimus Thoracis in Alentejana Breed from Divergent Growth Pathways. Animal.

[23] Jurie C., Martin J.F., Listrat A., Jailler R., Culioli J., Picard B. (2005). Effects of Age and Breed of Beef Bulls on Growth Parameters, Carcass and Muscle Characteristics. Anim. Sci..

[24] Jurie C., Martin J.F., Listrat A., Jailler R., Culioli J., Picard B. (2006). Carcass and Muscle Characteristics of Beef Cull Cows between 4 and 9 Years of Age. Anim. Sci..

[25] Picard B., Duris M.P., Jurie C. (1998). Classification of Bovine Muscle Fibres by Different Histochemical Techniques. Histochem. J..

[26] McGilchrist P., Greenwood P.L., Pethick D.W., Gardner G.E. (2016). Selection for Increased Muscling in Angus Cattle Did Not Increase the Glycolytic Potential or Negatively Impact PH Decline, Retail Colour Stability or Mineral Content. Meat Sci..

[27] Hwang Y.H., Kim G.D., Jeong J.Y., Hur S.J., Joo S.T. (2010). The Relationship between Muscle Fiber Characteristics and Meat Quality Traits of Highly Marbled Hanwoo (Korean Native Cattle) Steers. Meat Sci..

[28] Fry A.C. (2004). The Role of Resistance Exercise Intensity on Muscle Fibre Adaptations. Sport. Med..

[29] Gangnat I.D.M., Leiber F., Dufey P.A., Silacci P., Kreuzer M., Berard J. (2017). Physical Activity, Forced by Steep Pastures, Affects Muscle Characteristics and Meat Quality of Suckling Beef Calves. J. Agric. Sci..

[30] Vestergaard M., Oksbjerg N., Henckel P. (2000). Influence of Feeding Intensity, Grazing and Finishing Feeding on Muscle Fibre Characteristics and Meat Colour of Semitendinosus, Longissimus Dorsi and Supraspinatus Muscles of Young Bulls. Meat Sci..

[31] Brandstetter A.M., Picard B., Geay Y. (1998). Muscle Fibre Characteristics in Four Muscles of Growing Male Cattle II. Effect of Castration and Feeding Level. Livest. Prod. Sci..

[32] Maltin C.A., Lobley G.E., Grant C.M., Miller L.A., Kyle D.J., Morgan G.W., Matthews K.R., Sinclair K.D. (2001). Factors Influencing Beef Eating Quality 2. Effects of Nutritional Regimen and Genotype on Muscle Fibre Characteristics. Anim. Sci..

[33] Cassar-Malek I., Hocquette J.F., Jurie C., Listrat A., Jailler R., Bauchart D., Briand Y., Picard B. (2004). Muscle-Specific Metabolic, Histochemical and Biochemical Responses to a Nutritionally Induced Discontinuous Growth Path. Anim. Sci..

[34] Francisco C.L., Jorge A.M., Dal-Pai-Silva M., Carani F.R., Cabeço L.C., Silva S.R. (2011). Muscle Fiber Type Characterization and Myosin Heavy Chain (MyHC) Isoform Expression in Mediterranean Buffaloes. Meat Sci..

[35] Wegner J., Albrecht E., Fiedler I., Teuscher F., Papstein H.J., Ender K. (2000). Growth- and Breed-Related Changes of Muscle Fiber Characteristics in Cattle. J. Anim. Sci..

[36] Schreurs N.M., Garcia F., Jurie C., Agabriel J., Micol D., Bauchart D., Listrat A., Picard B. (2008). Meta-Analysis of the Effect of Animal Maturity on Muscle Characteristics in Different Muscles, Breeds, and Sexes of Cattle. J. Anim. Sci..

[37] Picard B., Jurie C., Duris M.P., Renand G. (2006). Consequences of Selection for Higher Growth Rate on Muscle Fibre Development in Cattle. Livest. Sci..

[38] Das R., Gupta I.D., Verma A., Singh S., Chaudhari M.V., Sailo L., Verma N., Kumar R. (2017). Single Nucleotide Polymorphisms in ATP1A1 Gene and Their Association with Thermotolerance Traits in Sahiwal and Karan Fries Cattle. Indian J. Anim. Res..

[39] Murphy K.T., Petersen A.C., Goodman C., Gong X., Leppik J.A., Garnham A.P., Cameron-Smith D., Snow R.J., McKenna M.J. (2006). Prolonged Submaximal Exercise Induces Isoform-Specific Na +-K+-ATPase MRNA and Protein Responses in Human Skeletal Muscle. Am. J. Physiol..

[40] Mberema C.H.H., Lietz G., Kyriazakis I., Sparagano O.A.E. (2016). The Effects of Gender and Muscle Type on the MRNA Levels of the Calpain Proteolytic System and Beef Tenderness during Post-Mortem Aging. Livest. Sci..

[41] Thomson B.C., Muir P.D., Dobbie P.M. (1999). Effect of Growth Path and Breed on the Calpain System in Steers Finished in a Feedlot. J. Agric. Sci..

[42] Coria M.S., Reineri P.S., Pighin D., Barrionuevo M.G., Carranza P.G., Grigioni G., Palma G.A. (2020). Feeding Strategies Alter Gene Expression of the Calpain System and Meat Quality in the Longissimus Muscle of Braford Steers. Asian-Australas. J. Anim. Sci..

[43] Lu Y., Bradley J.S., McCoski S.R., Gonzalez J.M., Ealy A.D., Johnson S.E. (2017). Reduced Skeletal Muscle Fiber Size Following Caloric Restriction Is Associated with Calpain-Mediated Proteolysis and Attenuation of IGF-1 Signaling. Am. J. Physiol..

